# Hyperhomocysteinemia and dyslipidemia in point mutation G307S of cystathionine β-synthase-deficient rabbit generated using CRISPR/Cas9

**DOI:** 10.1186/s12944-020-01394-5

**Published:** 2020-10-14

**Authors:** Ting Zhang, Rui Lu, Yibing Chen, Yuguo Yuan, Shaozheng Song, Kunning Yan, Yiwen Zha, Wenwen Zhuang, Yong Cheng, Jingyan Liang

**Affiliations:** 1 College of Veterinary Medicine, Yangzhou University, Yangzhou, 225009 Jiangsu China; 2 Jiangsu Co-innovation Center for Prevention and Control of Important Animal Infectious Diseases and Zoonoses, Yangzhou, 225009 Jiangsu China; 3 School of Pharmacy, Jiangsu Food & Pharmaceutical Science College, Huaian, 223003 Jiangsu China; 4 School of Nursing, Taihu University of Wuxi, Wuxi, 214000 Jiangsu China; 5 Institute of Translational Medicine, Medical College, Yangzhou University, Yangzhou, 225001 Jiangsu China

**Keywords:** Cystathionine β-synthase, Hyperhomocysteinemia, Dyslipidemia, Rabbits, CRISPR/Cas9, G307S mutation

## Abstract

**Background:**

Congenital hyper-homocysteinemia (HHcy) is caused by a defective cystathionine β-synthase (*CBS*) gene, and is frequently associated with dyslipdemia. The aim of this study was to further elucidate the effect of mutated *CBS* gene on circulating lipids using a rabbit model harboring a homozygous G307S point mutation in *CBS.*

**Methods:**

CRISPR/Cas9 system was used to edit the *CBS* gene in rabbit embryos. The founder rabbits were sequenced, and their plasma homocysteine (Hcy) and lipid profile were analyzed.

**Results:**

Six *CBS*-knockout (*CBS*-KO) founder lines with biallelic modifications were obtained. Mutation in *CBS* caused significant growth retardation and high mortality rates within 6 weeks after birth. In addition, the 6-week old *CBS*-KO rabbits showed higher plasma levels of Hcy, triglycerides (TG), total cholesterol (TC) and low-density lipoprotein cholesterol (LDL-C) compared to the age-matched wild-type (WT) controls. Histological analysis of the mutants showed accumulation of micro-vesicular cytoplasmic lipid droplets in the hepatocytes. However, gastric infusion of vitamin B and betaine complex significantly decreased the plasma levels of TG, TC and LDL-C in the *CBS*-KO rabbits, and alleviated hepatic steatosis compared to the untreated animals.

**Conclusion:**

A *CBS*^G307S^ rabbit model was generated that exhibited severe dyslipidemia when fed on a normal diet, indicating that G307S mutation in the *CBS* gene is a causative factor for dyslipidemia.

## Introduction

Homocysteine (Hcy) is a non-essential sulphur-containing amino acid derived from methionine metabolism. The plasma Hcy range in healthy individuals is 5–15 μmol/L, and an increase to 16–30 μmol/L, 31–100 μmol/L and > 100 μmol/L result in moderate, intermediate and severe hyper-homocysteinemia (HHcy) respectively [1]. Moderate HHcy is present in 5–7% of the general population, and increases the risk of fatty liver, diabetes, atherosclerosis [2–5], atherothrombosis, stroke, ischemic heart disease and peripheral vascular disease [6–11]. In addition, a 2.5 μmol/L increase in Hcy increases the risk of cardiovascular diseases (CVDs) by 10% [12], indicating its potential as a biomarker of coronary heart failure [9, 13, 14].

The causative factors of HHcy include genetics, nutrition, medication, disease status, smoking and age. In addition, severe HHcy is often triggered by congenital deficiency of cystathionine β-synthase (*CBS*) or 5,10-methylenetetrahydrofolate reductase (*MTHFR*) [15], and the most frequent cause is a rare autosomal recessive mutation in the *CBS* gene. In fact, around 22 mutant alleles of *CBS* have been reported so far in individuals with *CBS* deficiency [16–22], and 10 missense mutations including G307S, I278T, V320A, T353M, L101P, A226T, N228S, A231L, D376N and Q526K have been identified by DNA sequencing [23]. The most common mutations are I278T and G307S from exon 8. One study reported 21% prevalence of the G307S mutation in UK, 8% in US patients and 71% in Ireland [24].

Hcy is re-methylated to methionine by 5-methyltetrahydrofolate-homocysteine methyltransferase (MHMT) and the cofactor folate-cobalamin, and the methyl group is donated by betaine via betaine-homocysteine methyl transferase (BHMT). In addition, *CBS* metabolizes Hcy to cystathionine in the presence of vitamin B6 [25–30]. Interestingly, there is considerable clinical heterogeneity among individuals with homocystinuria based on their responsiveness to pyridoxine (vitamin B6) [31]. While patients harboring the G307S mutation are completely non-responsive, those with I278T partially respond to B6. In addition, pyridoxine resistance is associated with a more severe clinical phenotype [20]. Although the precise mechanism through which G307S affects CBS function is unclear, there is evidence that it restricts the ability of tyrosine at position 308 to assume the proper conformational state required for the pyridoxial-cystathionine intermediate, eventually inhibiting the catalytic performance [32]. In addition, the pyridoxine non-responsive patients may benefit from betaine supplementation [33], either alone or in combination with vitamin B12, folic acid or a methionine-restricted diet.

HHcy is routinely accompanied by life-threatening vascular complications, and individuals with high circulating levels of Hcy are at a higher risk of CVD and increased 5-year mortality [34]. However, most animal models currently used for cardiovascular research do not accurately simulate the human system [35]. For instance, most disease-causing mutations in humans do not replicate the symptoms in rodents, and their short life-span precludes any investigation of long-term effects. Furthermore, rats, mice, tree shrews and dogs are resistant to atherosclerosis and hypercholesterolemia, unlike primates, hamsters and rabbits [36–39]. The rabbit (*Oryctolagus cuniculus*) model offers several advantages over rodents, such as greater phylogenetic similarity to primates, adequate amount of blood for plasma biochemical analysis, suitable heart size for studying atherosclerosis in both aorta and coronary arteries [35, 40], and a more diverse genetic background which is conducive to modelling complex diseases and simulating the effects of genetic diversity in the human population [35].

Since most *CBS* mutations in humans are of the missense type caused by base pair substitutions, HHcy modeling require gene knock-in as opposed to knock-out. To this end, we generated *CBS* gene mutant rabbits including point mutation G307S using the CRISPR/Cas9 system in order to gain new insights into the HHcy pathogenesis. In addition, the *CBS*-KO rabbits were fed with vitamin B and betaine complex supplements to devise possible therapeutic approaches.

## Material and methods

### Construction of the CRISPR/Cas9 system

**Fig. 1:**
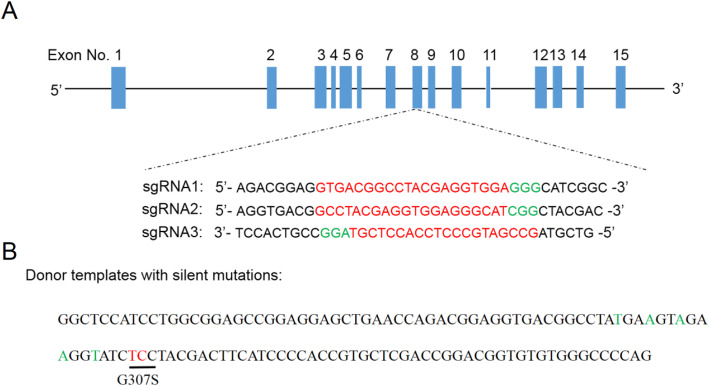
Homology-dependent repair (HDR) knock-in of *CBS* gene in rabbits using CRISPR/Cas9 gene editing. **a** CRISPR/Cas9-targeting sites on the rabbit *CBS* gene. The red letters in the sgRNA sequences are specific for exon 8, and the green letters indicate the protospacer adjacent motif (PAM). **b** Donor template sequences with silent mutations (green letters) and G307S (red letters)

The CRISPR/Cas9 single guide RNAs (sgRNAs) for rabbit *CBS* were designed using http://crispr.mit.edu based on its sequence (Genbank: NW_003160195.1; http://www.ncbi.nlm.nih.gov/). Three *CBS*-targeting sgRNAs were screened (sgRNA1 sited E8: g.6631–6650, sgRNA2 sited E8: g.6637–6656 and sgRNA3 sited E8: 6641–6660) (Fig. 1). The detailed protocol has been described previously [41, 42]. Single-stranded oligodeoxynucleotide donor templates (ssODN) with silent mutations were designed and synthesized by Shanghai Sangon Biotechnology Co. Ltd. as follows: GGCTCCATCCTGGCG GAGCCGGAGGAGCTGAA CCAGACGGAGGTGACGGCCTAtGAaGTaGAaGGtATC tcCTACGACTTCATC CCCACCGTGCTCGAC CGGACGGTGTGT GGGCCCCAG.

### Zygote injection with Cas9/sgRNA and embryo transfer

New Zealand White rabbits (6–8 months old) were housed at the Animal Genetic Engineering Laboratory of Yangzhou University under a 12 h diurnal/nocturnal cycle, and fed twice a day with free access to water. All protocols were approved by the Care and Use of Laboratory Animals (Ministry of Science and Technology of the People’s Republic of China) and the Animal Care and Use Committee of Yangzhou University, Yangzhou, China (license number: SYXK(Su)2017–0044).

Female rabbits were superovulated by intramuscular injection of follicle-stimulating hormone (Sansheng Pharmaceutical Co., Ltd., NingBo, China) twice daily. The dosage was 15 IU for the first two injections, 10 IU for the next two and 5 IU for the last two injections. After the final injection, both the superovulated and recipient females were injected with 100 IU human chorionic gonadotropin (HCG), and the former were mated with male rabbits. Approximately 18–20 h post-coitus, the female rabbits were anesthetized with pentobarbital (2%, 20 mg/kg, i.p.), and the pronuclear zygotes were extracted and transferred into M2 medium (Sigma-Aldrich,

St. Luis, MO, USA) supplemented with 10% fetal bovine serum (Hyclone, Logan, UT, USA). Following cytoplasmic injection of 40 ng/μL Cas9 mRNA, 10 ng/μL of an sgRNA and 25 ng/μL ssODN, the embryos were transferred to complete (with 10% FBS) M16 medium (Sigma-Aldrich, St. Luis, MO, USA) and incubated at 38 °C under 5% CO_2_ for 30–60 min. Approximately 15–20 embryos were transferred to one recipient female.

### Genotypic analysis

Genomic DNA was extracted from ear biopsies via phenol-chloroform extraction, and the *CBS* sequences were amplified using specific primers (Table 1). The amplified products were extracted and purified from the gel using a PCR Purification kit (Transgene Biotechnology Co., Ltd., Beijing, China). The sequences were then cloned into pGEM-T vector (Promega, Madison, WI, USA), and sequenced by Shanghai Sangon Biotechnology Co. Ltd. Sequences were analyzed by the Lasergene DNA analysis package (DNAStar Inc., Madison, WI, USA).

**Table 1:** Primers for detection of *CBS -*KO rabbits

Name	5′-3’	TM
CBS-1	GCTGGCTTCAGTCAGGTTC	55.1 °C
CBS-2	CTCCTTGTCGGTGCTCTTG	56.1 °C

To determine any off-target effects, the CRISPR design tool (http://tools.genomeengineering.org) was used to predict potential sites homologous to the 23-bp sgRNA + PAM sequence across the rabbit genome. These sites were then amplified in the genomic DNA of founder *CBS*-KO rabbits and sequenced. The off-target sites and primer pairs are listed in Supplementary Tables 1 and 2 (Table S1, Table S2).

### Drug test

Two weeks-old *CBS*-KO and WT rabbits were infused daily with 2.5 mg/kg vitamin B6, 25 μg/kg vitamin B12, 45 μg/kg folate and 25 mg/kg betaine via the gastric route. The animals were acclimatized to gentle restraint and handling, and infused daily from 10 am to 12 noon for 4 weeks by the same technician.

### Biochemical analysis

Four milliliters peripheral blood was collected into EDTA-coated tubes from each animal after withholding food for 10–12 h. Plasma was separated by centrifuging the blood at 3000 rpm at 4 °C. The levels of triglycerides (TG), total cholesterol (TC), and high-density (HDL-C) and low-density lipoprotein cholesterol (LDL-C) were measured using specific kits (A110–1, A111–1, A112–1 and A113–1; Nanjing Jiancheng Bioengineering Institute, Nanjing, China). In addition, Hcy levels were measured using an ELISA kit (Laier Biotechnology Co., Ltd., Hefei, China).

### Western blotting

The expression levels of the apolipoproteins (Apo) B, E and A-I in the plasma were analyzed by Western blotting as per standard protocols. The protein bands were probed with the goat anti-ApoE and anti-ApoB (both from Rockland, Limerick PA), and sheep anti-ApoA-I (AbD Serotec, Oxford, UK) antibodies. The secondary antibodies were HRP-conjugated donkey anti-goat IgG (Jackson Immuno Research Laboratories, West Grove, PA, US) and donkey anti-sheep IgG (Chemicon, Temecula, CA, US).

### Histomorphological assessment

The *CBS*-KO and WT littermates were maintained under similar conditions, and fed according to their growth stage. The body weight of the rabbits was monitored from birth till 6 weeks of age. After euthanizing the animals with sodium pentobarbital overdose, the liver lobes were harvested and fixed in 4% paraformaldehyde. For histological analysis, the fixed tissues were embedded in paraffin and cut into sections that were stained using hematoxylin and eosin (HE) using standard protocols. The stained sections were viewed under a Leica light microscope.

### Statistical analysis

Data were expressed as mean ± SD and compared by Student’s t test. GraphPad Prism was used for all statistical analyses, and *P* < 0.05 was considered statistically significant.

## Results

### Generation of *CBS*-KO rabbits and genotypic analysis

**Fig. 2:**
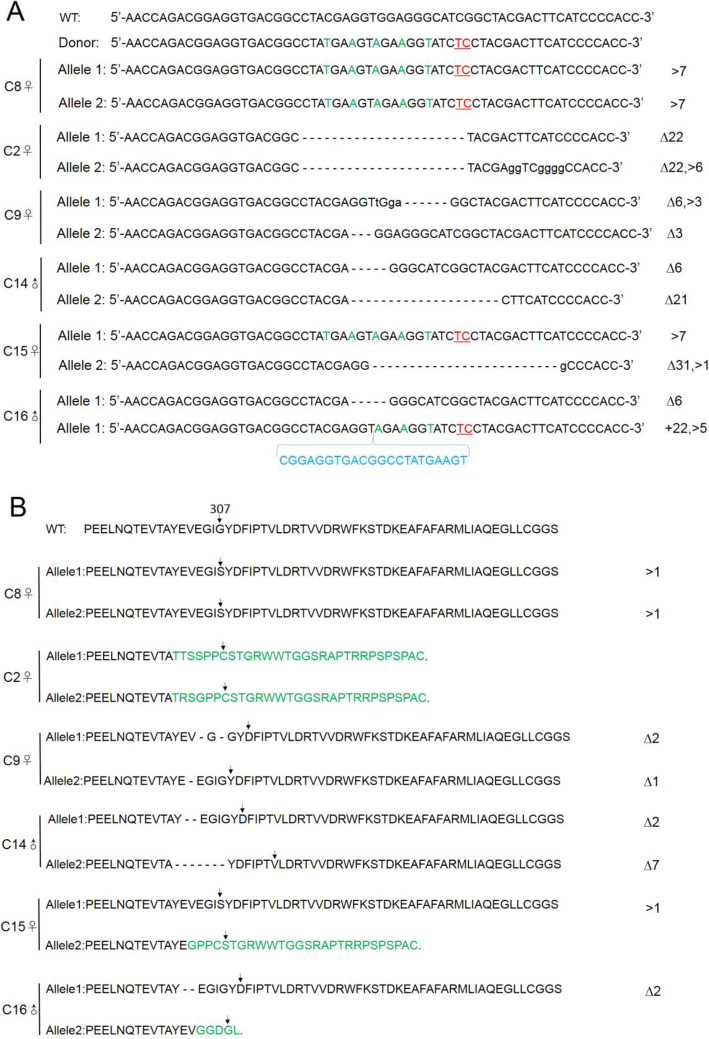
Generation of mutant *CBS* alleles by injecting rabbit zygotes with CRISPR/Cas9. **a** Types of mutations in the *CBS* alleles in founder rabbits. The WT and donor ssODN template sequences are shown at the top. Silent mutations, point mutations, substitutions (>) and insertions (+) are labeled with green letters, red underlined letters, black lowercase letters and blue letters respectively. The deletions (∆) are indicated on the right of the respective allele. **b** Theoretical amino acid sequences of the founder rabbits. The amino acid at 307 is shown with a black arrow. Deletions (∆) and substitutions (>) are shown to the right of each allele

As shown in Table 2, we injected 217 zygotes with the CRISPR/Cas9 and sgRA constructs, and transferred 181 into 9 surrogate females. Eighteen pups harbored a mutant *CBS* (Table 2), of which only 6 survived and were numbered C2♀, C8♀, C9♀, C14♂, C15♀ and C16♂. C8♀ was homozygous for G307S, C2♀ harbored a frameshift mutation in *CBS* due to deletion or substitution, and C9♀, C14♂ and one allele of C16♂ harbored deletions/substitutions in the *CBS* gene without a frameshift in the amino acid sequence. The second *CBS* allele of C16♂ had deletions/insertions resulting in a frameshift mutation. One allele of C15♀ harbored the G307S and the other showed deletions/insertions resulting in frameshift mutation (Fig. 2). In addition, 15 potential off-target sites (OTs, five for each sgRNA) were also amplified and sequenced, and no overlapping peaks were detected near the OTs.

**Table 2:** Generation of *CBS*-KO rabbits using CRISPR/Cas9

Number of injected zygotes	217
Number of transferred zygotes	181
Number of recipients	9
Number of pregnancies	5
Total births	18
Number of mutants	18
Number of G307S mutants	1
Pregnancy rate	55.56%
Mutation rate	100%
G307S rate	5.56%

### Mutations in *CBS* induced hyperlipidemia which was reversed by betaine

Since HHcy is closely associated with dyslipidemia, we next analyzed the levels of blood lipids in the WT and mutant rabbits. As shown in Table 3, the C2♀ and C8♀ rabbits fed with normal chow had significantly higher serum levels of Hcy, TG, TC and LDL-C compared to the age-matched WT rabbits. The Hcy levels in C2♀ (48.80 μmol/L) and C8♀ (52.66 μmol/L) were almost twice as high as that in WT controls, whereas the TG levels (3614.93 mg/dL and 3878.47 mg/dL respectively) showed a 50–54 fold increase. The TC levels in C2♀ (738.98 mg/dL) and C8♀ (342.62 mg/dL) were respectively 6- and 3-fold higher, and that of LDL-C levels in both (67.24 mg/dL and 76.80 mg/dL respectively) were 2-fold higher compared to the WT. In contrast, the HDL-C level in C8♀ (16.64 mg/dL) was significantly lower compared to the WT. Consistent with the high levels of circulating lipids in C8♀, the color of its plasma was milky white (Fig. 4b).

**Table 3:** Plasma Hcy and lipid profile of the rabbits without vitamin B and betaine supplementation

Cholesterol	Hcy (μmol/L)	TG (mg/dL)	TC (mg/dL)	HDL-C (mg/dL)	LDL-C (mg/dL)^a^
WT(*n* = 6)	27.93 ± 3.49	71.31 ± 13.39	122.89 ± 23.66	38.53 ± 1.73	28.25 ± 7.09
C2♀	48.80	3614.93	738.98	91.33	67.24
C8♀	52.66	3878.47	342.62	16.64	76.80

Values are expressed as means ± SD.^a^The data does not contain vLDL-C

**Fig. 3:**
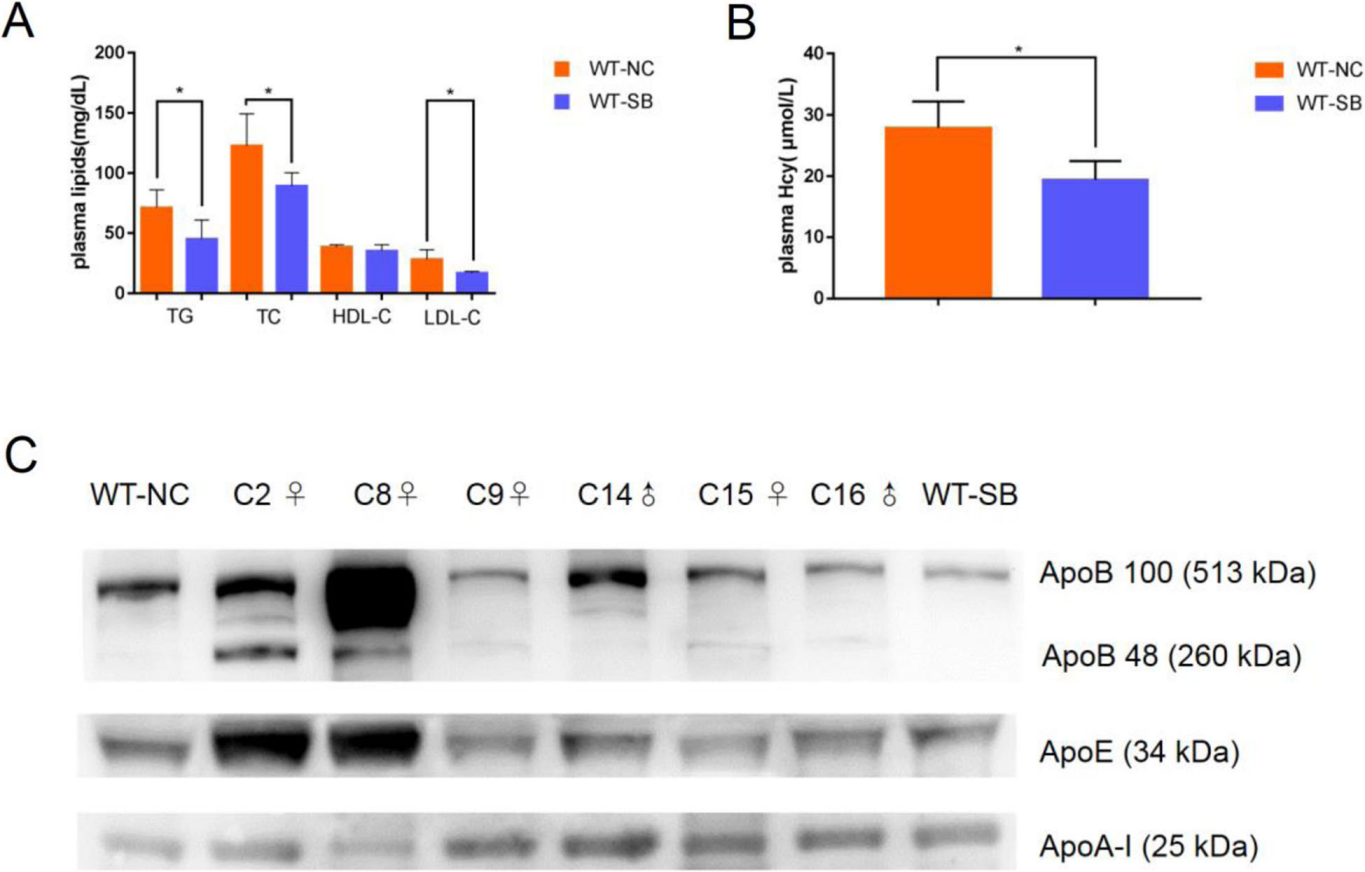
*CBS*-KO rabbits are hyperlipidemic. **a** Plasma TG, TC, HDL-C, LDL-C and **b** Hcy levels in 6 weeks-old WT rabbits (*n* = 6). Mean ± SD, *P < 0.05. **c** Immunoblot showing levels of plasma apolipoproteins in the indicated groups. WT-NC: wild type rabbits fed normal chow. WT-SB: wild type rabbits fed normal chow supplemented with the vitamin B and betaine complex. C2♀, C8♀: *CBS*-KO rabbits on a normal chow diet. C9♀, C14♂, C15♀, C16♂: *CBS*-KO rabbits fed normal chow supplemented with the vitamin B and betaine complex

To evaluate a potential hypolipidemic effect of betaine, we infused both WT and mutant animals with vitamin B and betaine complex for 4 weeks. Betaine supplementation reduced plasma Hcy, TG, TC and LDL-C by 30.50% (19.41 ± 1.77 μmol/L vs 27.93 ± 2.47 μmol/L), 36.88% (45.00 ± 7.16 mg/dL vs 71.31 ± 6.70 mg/dL), 27.31% (89.33 ± 4.94 mg/dL vs 122.89 ± 11.83 mg/dL) and 39.33% (17.14 ± 0.56 mg/dL vs 28.25 ± 3.54 mg/dL) respectively in the WT rabbits compared to non-supplemented littermates. In contrast, no significant changes were seen in plasma HDL-C levels (Fig. 3a). The similarly fed C9♀, C14♂, C15♀ and C16♂ mutants showed a significant decrease in plasma levels of TG, TC, LDL-C and Hcy compared to that in C2♀ and C8♀. In addition, the TG levels in C14♂ (76.14 mg/dL) and C15♀ (35.95 mg/dL), and the LDL-C levels in C16♂ (24.64 mg/dL) dropped to near normal range after betaine supplementation (Tables 3 and 4). Consistent with these observations, the C2♀ and C8♀ rabbits showed a significant increase in ApoE and ApoB compared to the WT, whereas betaine supplementation in C9♀, C14♂, C15♀ and C16♂ reversed these trends (Fig. 3c).

**Table 4:** Plasma Hcy and lipid profile of the rabbits with vitamin B and betaine supplementation

Cholesterol	Hcy (μmol/L)	TG (mg/dL)	TC (mg/dL)	HDL-C (mg/dL)	LDL-C (mg/dL)^a^
WT(n = 6)	19.41 ± 2.50	45.00 ± 14.35	89.33 ± 9.88	35.24 ± 4.57	17.14 ± 1.11
C9♀	26.40	103.27	36.55	3.48	15.35
C14♂	25.06	76.14	293.89	19.48	88.02
C15♀	25.25	35.95	160.09	27.48	61.66
C16♂	30.8	22.03	61.87	23.22	24.64

Values are expressed as means ± SD.^a^The data does not contain vLDL-C

### The morphology of *CBS*-KO rabbits

**Fig. 4:**
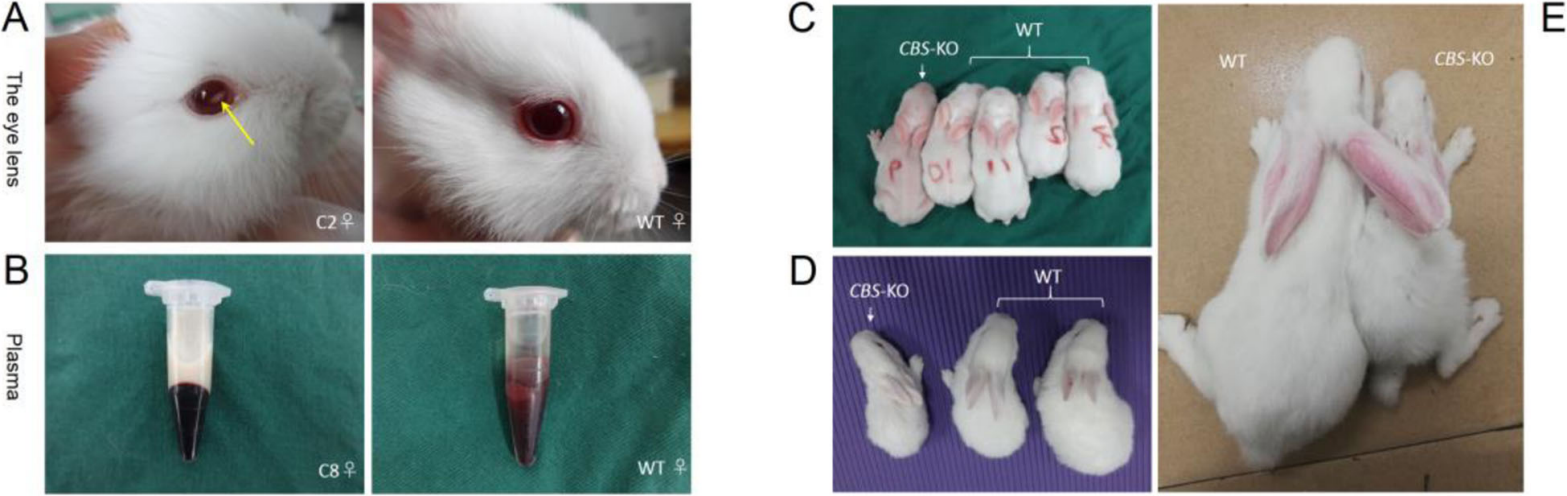
The *CBS*-KO rabbits showed significant growth retardation. **a** Representative images of the eyes indicating lens dislocation (yellow arrow) in the C2♀ rabbit. **b** Representative pictures of the milky white plasma of C8♀ (G307S) and transparent plasma of WT rabbits. Representative images of the *CBS*-KO and WT rabbits at (**c**) 1 week, (**d**) 3 weeks and (**e**) 9 weeks

The body weights of the WT and *CBS*-KO rabbits were similar on postnatal days 1 and 7. At 3 weeks however, the *CBS*-KO rabbits weighed significantly less compared to the WT animals (Table 5), and some failed to gain weight even after 6 weeks. In addition, *CBS*-KO rabbits were overall smaller, and had sparser fur and pale mucous membranes (Fig. 4c-e), all of which are indicative of growth retardation. The eye-lens were also dislocated in the *CBS*-KO rabbits (Fig. 4a). Finally, most of the mutant animals did not survive beyond 5 ~ 7 weeks after birth (Table 6).

**Table 5:** Growth of *CBS*-KO and WT rabbits from 1 to 42 days old

Postpartum age, days	Body weight, g							
	C2♀	C8♀	C9♀	C24♂	C25♀	C26♂	*CBS*-KO (*n* = 6)	WT(*n* = 6)
1	58	67	60	52	61	59	59.50 ± 4.43	55.17 ± 3.28
7	73	84	83	77	88	68	78.83 ± 6.87	88.33 ± 6.97
14	123	112	124	102	113	118	115.33 ± 7.48*	133.17 ± 15.24
21	161	176	177	129	169	165	162.83 ± 16.15**	208.6 ± 22.48
28	214	216	234	205	240	236	224.17 ± 13.06**	278.3 ± 30.60
35	310	306	300	302	350	353	320.17 ± 22.39**	359.5 ± 28.58
42	382	397	425	413	438	453	418 ± 23.93****	590.6 ± 40.86

Values are expressed as means ± SD. *P < 0.05, **P < 0.01 and ****P < 0.0001

**Table 6:** The survival duration of CBS-KO rabbits

	C2♀	C8♀	C9♀	C24♂	C25♀	C26♂
days	46	43	50	66	109	52

### Histological analysis

**Fig. 5:**
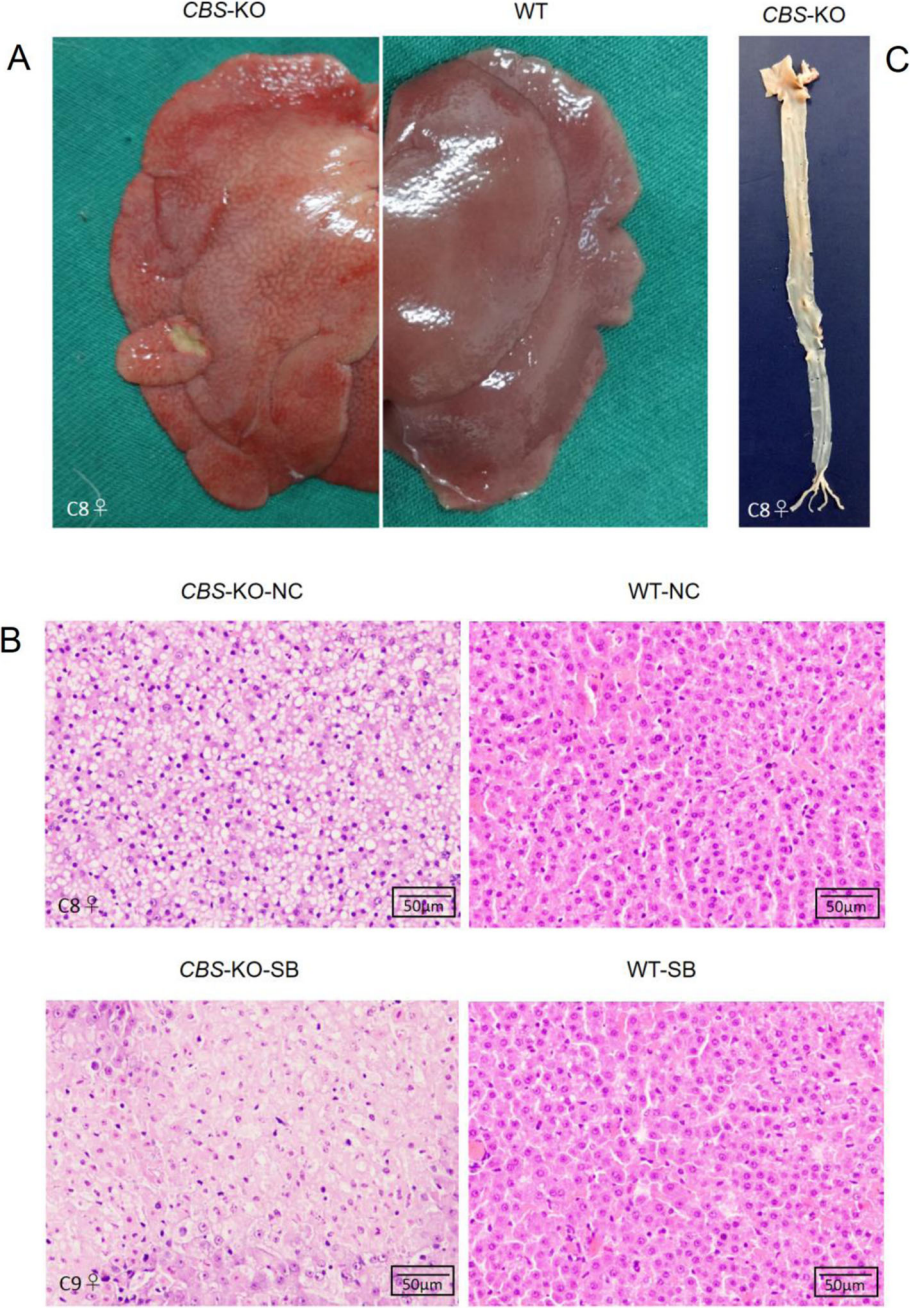
*CBS*-KO rabbits showed signs of fatty liver. **a** Representative images of the liver from *CBS*-KO (C8) and WT rabbits. **b** Representative images of HE-stained liver sections from the indicated groups. *CBS*-KO-NC: *CBS*-KO rabbits (C8♀) fed normal chow. WT-NC: wild type rabbits fed normal chow. *CBS*-KO-SB: *CBS*-KO rabbits (C9♀) fed normal chow supplemented with vitamin B and betaine complex. WT-SB: wild type rabbits fed normal chow diet supplemented with vitamin B and betaine complex. Magnification – 200x; Scale bar = 50 μm. **c** Gross lesions of aortic atherosclerosis in *CBS*-KO rabbit (C8♀, 6 weeks) stained with Sudan IV

The 6-week-old WT and *CBS*-KO rabbits receiving normal and vitamin B/betaine complex-supplemented feed were sacrificed for histological examination. The livers of *CBS*-KO rabbits were brick red compared to the reddish-brown color of the WT livers (Fig. 5a).

Histologically, no cytoplasmic lipid droplets were observed in the liver of WT rabbits even in the absence of vitamin B and betaine complex supplementation. However, the *CBS*-KO rabbits had significant accumulation of macro cytoplasmic lipid droplets in their liver, which decreased following vitamin B and betaine complex supplementation (Fig. 5b). However, Sudan IV staining did not reveal any atheromatous plaques in the aorta and other vascular segments of the *CBS*-KO rabbits (Fig. 5c).

## Discussion

Homocysteinemia is observed in 5–30% of the human population, and leads to vascular complications in one-fourth of the patients [43–45]. We obtained 6 *CBS*-KO rabbits including one harboring the G307S point mutation, using the CRISPR/Cas9 gene editing technology. All mutants (C2♀ and C8♀) lacked the glycine 307 residue in CBS protein, and displayed high serum levels of Hcy and lipids. Supplementing the normal chow with vitamin B and betaine alleviated the pathological symptoms of HHcy. Although there was some research reported on the G307S point mutation, it was mostly based on the heterologous gene expression system such as *E. coli*, *S. cerevisiae*, and Chinese hamster ovary cells. But it was unfortunate that the heterologous CBS^G307S^ protein lacked enzyme activity [46–48]. At present, there was no *CBS-*deficient animal model of G307S point mutation or deletion of glycine at position 307. The *CBS*-KO rabbit showed HHcy, dyslipidemia, a short life span, hepatic steatosis and other lesions. This indicates that HHcy and hyperlipidemia are likely the result of 307G deletion as well as G → S mutation. Compared with *CBS*-deficient mice in the previous research [49, 50], the results of this study may be more precise and accurate in explaining the molecular mechanism of hereditary hyperhomocysteinemia etiology. Therefore, the *CBS*-KO rabbit model is suitable for studying the pathogenesis and therapeutic strategies of HHcy. Studies show that HHcy is an independent risk factor for CVDs [3], which correlates positively with atherosclerotic symptoms induced by the high levels of TG, TC and LDL-C, and normal plasma HDL-C level [51, 52]. In addition, HHcy increases the risk of hypertriglyceridemia [53, 54]. Consistent with this, the serum triglyceride levels were high in the *CBS*-KO founder rabbits, and decreased significantly following betaine and vitamin B6 supplementation.

Dyslipidemia is a major factor underlying CVDs, and normalizing blood lipid levels can lower the morbidity and mortality in patients with heart disease [55]. The quaternary amine betaine and B vitamins are efficient methyl donors that produce a large amount of free carnitine in the liver, which promotes long-chain ester acylCoA entry into the mitochondria and accelerates fatty acid oxidation, eventually reducing the blood lipid levels [56]. Betaine may inhibit hepatic biosynthesis of LDL-C or accelerate the conversion of LDL to HDL [57]. In addition, oral betaine and B vitamins can reduce serum Hcy levels in healthy individuals by increasing their metabolism, and have therapeutic effects on patients with hyperlipidemia, fatty liver and atherosclerosis [58–60]. Consistent with this, vitamin B and betaine complex supplementation significantly improved the blood lipid profile of both WT and *CBS*-KO rabbits. Interestingly, the normal rabbits also showed Hcy levels higher than 15 μmol/L, which may explain the rapid induction of hyperlipidemia via high-fat diet consumption. Thus, the species and nutrient intake should be taken into account when assessing the effect of Hcy metabolism on dyslipidemia.

The fatty liver parenchyma of *CBS-KO* rabbits was filled with microvesicular cytoplasmic lipid droplets due to the influence of Hcy on the expression level of S-methyltransferase (BHMT). *CBS*-KO rabbits showed signs of growth retardation and short survival. Betaine and vitamin B supplementation reduced the fatty liver symptoms and prolonged survival of the mutants. Maclean [61] et al. reported that transgenic expression of *CBS* alleviated liver steatosis and prolonged survival in a mouse model. We showed for the first time that the addition of betaine and B vitamins partly compensated for the lack of *CBS*. This is significant since the previously reported hypolipidemic effects of betaine were seen animal models with high-fat diet-induced hyperlipidemia.

HHcy is an important factor in hyperlipidemia and cardiovascular disease. In this study, animal models of HHcy showed hyperlipidemia and cardiovascular disease symptoms. Although the lipoprotein metabolism and cardiovascular pathophysiology of rabbits are similar to that of humans [62], the inherently higher Hcy levels in rabbits may have resulted in premature death in the absence of *CBS*.

Compared to the untreated animals, the *CBS*-KO rabbits with betaine and vitamin B supplementation had lower ApoB100, ApoB48 and ApoE levels in their plasma. ApoB is the major component of very low density lipoprotein (vLDL) and LDL particles. Namekata et al. [49] detected elevated ApoB in CBS^−/−^ mouse serum, and Salahi et al. [63] found that betaine supplementation decreased LDL concentration and increased that of HDL in a rat model. ApoB is abundant in human and rabbit but not murine plasma [64]. In addition, the chemical composition and cholesterol ester transfer subunits of human and rabbit ApoB-related lipoproteins are similar, which correlates with the role of rabbit ApoB in atherosclerosis [65].

In conclusion, CBS-KO rabbits have impaired growth and metabolism, although it remains to elucidated whether these changes are the direct effect of the mutation, or an indirect effect of hyperlipidemia or high homocysteine.

## Study strengths and limitations

We have generated *CBS*-KO rabbits with G307S mutation for the first time using the CRISPR/Cas9 system, and the model exhibits hyperhomocysteinemia and dyslipidemia on a normal chow diet. It is also the first report on the effect of betaine on the serum lipids of CBS-KO rabbits.

However, the short lifespan of these rabbits obviated the control data of C9♀, C14♂, C15♀ and C16♂ without betaine and vitamin B complex supplementation. Nevertheless, Hcy, TG, TC, LDL-C and other indices of most *CBS*-KO rabbits were higher compared to that of the normal controls even after betaine and vitamin B complex supplementation.

## Conclusion

In conclusion, the *CBS* mutated rabbit model is a promising tool for studying human dyslipidemia, despite their high mortality. The lesions of G307S homozygous *CBS*-KO rabbit should be useful to clearly understand the pathophysiology of the *CBS* gene deficiency and the G307S mutation rabbit provides a suitable animal model for drug development.

## Supplementary information

**Additional file 1: Table S1.** The sequences of potential off-target sites.

**Additional file 2: Table S2.** The sequences of potential off-target loci PCR primers.

## Abbreviations

HHcy: Hyper-homocysteinemia
CBS: Cystathionine β-synthase
TG: Triglycerides
TC: Total cholesterol
LDL-C: Low-density lipoprotein cholesterol
HDL-C: High-density lipoprotein cholesterol
vLDL-C: Very Low-density lipoprotein cholesterol
CVDs: Cardiovascular diseases
ApoB: Apolipoprotein B
APOE: Apolipoprotein E
