# Supplementary material for: Hyperhomocysteinemia and dyslipidemia in point mutation G307S of cystathionine β-synthase-deficient rabbit generated using CRISPR/Cas9
Source: Lipids Health Dis. 2020 Oct 14;19:224. doi: 10.1186/s12944-020-01394-5 (PMC7560309; doi:10.1186/s12944-020-01394-5)
Supplement: Supplementary file 1 — Additional file 1: Table S1. The sequences of potential off-target sites. [file 12944_2020_1394_MOESM1_ESM.docx]

**Table S1. The sequences of potential off-target sites.**

A

|  | 20 | 19 | 18 | 17 | 16 | 15 | 14 | 13 | 12 | 11 | 10 | 9 | 8 | 7 | 6 | 5 | 4 | 3 | 2 | 1 | N | G | G |
| --- | --- | --- | --- | --- | --- | --- | --- | --- | --- | --- | --- | --- | --- | --- | --- | --- | --- | --- | --- | --- | --- | --- | --- |
| sgRNA-1 | G | T | G | A | C | G | G | C | C | T | A | C | G | A | G | G | T | G | G | A | G | G | G |
| OT1 | T | T | T | G | C | G | G | C | C | T | A | C | G | A | G | G | T | G | A | A | G | G | G |
| OT2 | C | T | C | C | C | A | G | C | C | T | A | G | G | A | G | G | T | G | G | A | G | G | G |
| OT3 | T | C | C | C | T | G | A | C | C | T | A | C | T | A | G | G | T | G | G | A | G | G | G |
| OT4 | T | T | C | C | A | G | G | A | A | T | A | C | G | A | G | G | T | G | G | A | G | G | G |
| OT5 | T | A | A | G | C | T | G | C | C | T | A | C | C | A | G | G | T | G | G | A | G | G | G |

B

|  | 20 | 19 | 18 | 17 | 16 | 15 | 14 | 13 | 12 | 11 | 10 | 9 | 8 | 7 | 6 | 5 | 4 | 3 | 2 | 1 | N | G | G |
| --- | --- | --- | --- | --- | --- | --- | --- | --- | --- | --- | --- | --- | --- | --- | --- | --- | --- | --- | --- | --- | --- | --- | --- |
| sgRNA-2 | G | C | C | T | A | C | G | A | G | G | T | G | G | A | G | G | G | C | A | T | C | G | G |
| OT6 | G | G | C | A | G | C | T | G | G | G | T | G | G | A | G | G | G | C | A | T | C | G | G |
| OT7 | G | T | G | T | T | G | T | G | T | G | T | G | G | A | G | G | G | C | A | T | C | G | G |
| OT8 | A | T | C | G | C | T | G | T | G | C | T | G | G | A | G | G | G | C | A | T | C | G | G |
| OT9 | A | G | C | A | A | G | G | C | A | G | T | G | G | A | G | G | G | C | A | T | C | G | G |
| OT10 | A | C | G | C | C | G | C | A | G | G | T | G | C | A | G | G | G | C | A | T | C | G | G |

C

|  | 20 | 19 | 18 | 17 | 16 | 15 | 14 | 13 | 12 | 11 | 10 | 9 | 8 | 7 | 6 | 5 | 4 | 3 | 2 | 1 | N | G | G |
| --- | --- | --- | --- | --- | --- | --- | --- | --- | --- | --- | --- | --- | --- | --- | --- | --- | --- | --- | --- | --- | --- | --- | --- |
| sgRNA-3 | G | C | C | G | A | T | G | C | C | C | T | C | C | A | C | C | T | C | G | T | A | G | G |
| OT11 | T | T | G | C | A | A | G | C | C | C | T | C | C | A | C | C | T | C | C | T | A | G | G |
| OT12 | G | A | G | G | T | C | T | C | C | C | T | C | C | A | C | C | T | G | G | T | A | G | G |
| OT13 | A | G | C | C | A | G | C | C | C | C | T | C | C | G | C | C | T | C | G | T | A | G | G |
| OT14 | C | T | T | G | G | G | C | C | C | C | T | G | C | A | C | C | T | C | G | T | A | G | G |
| OT15 | G | G | C | C | T | G | G | A | C | A | T | C | C | A | C | C | T | C | G | T | A | G | G |

A) OT1 to OT5 homologous to sgRNA-1. B) OT6 to OT10 homologous to sgRNA-2. C) OT7 to OT15 homologous to sgRN
