# Supplementary material for: Hyperhomocysteinemia and dyslipidemia in point mutation G307S of cystathionine β-synthase-deficient rabbit generated using CRISPR/Cas9
Source: Lipids Health Dis. 2020 Oct 14;19:224. doi: 10.1186/s12944-020-01394-5 (PMC7560309; doi:10.1186/s12944-020-01394-5)
Supplement: Supplementary file 2 — Additional file 2: Table S2. The sequences of potential off-target loci PCR primers. [file 12944_2020_1394_MOESM2_ESM.docx]

**Table S2. The sequences of potential off-target loci PCR primers.**

| **Primers** | **Primer sequences** |
| --- | --- |
| OT-1 | 5′- TAGAAAGTCCCGAGGTTGTG -3′  5′- GGATGGGTGAATAAATGTGC -3′ |
| OT-2 | 5′- TCGTTCGGTTAGCATT -3′  5′- AAGACAGCCTACATCCC -3′ |
| OT-3 | 5′- TGATAGGGAAGACAGG -3′  5′- TAGGGATAGAGCCAGA -3′ |
| OT-4 | 5′- TGGGAAGGCGGAGAAA -3′  5′- GGCGTGATGCTGGAAA -3′ |
| OT-15 | 5′- ATTCCCACAAATCACA -3′  5′- GTCAGCCCACCCGTAA-3′ |
| OT-6 | 5′- TGGGAGGCGGAGCAAGT -3′  5′- TGACCAAAGCCGAGAAC -3′ |
| OT-7 | 5′- CTCGTATCCTTTCTGTCCA -3′  5′- AGTCCGCATCCTTTTCTG -3′ |
| OT-8 | 5′- CGGACAGCATACCATC -3′  5′- CCAAAACGAGGAAAAG -3′ |
| OT-9 | 5′- TCACAAGGTGGAAGAATC -3′  5′- GGCTCAAAGGAGGAAA -3′ |
| OT-10 | 5′- GGAGTGACCCAGAGGATG -3′  5′- GAGGCGAGGAAGAAAGAA -3′ |
| OT-11 | 5′- TCCTCTGTTGGCATTT -3′  5′- ACACTTGGGACTCTTTG -3′ |
| OT-12 | 5′- GTCAGCCCACCCGTAA -3′  5′- TTCCCACAAATCACATCC -3′ |
| OT-13 | 5′- GAAGTTCTTGAAGCGGTGGT -3′  5′- AGGCAGGTGGATGGGTG -3′ |
| OT-14 | 5′- ATTCCCACAAATCACA -3′  5′- GTCAGCCCACCCGTAA -3′ |
| OT-15 | 5′- GATGAGGTGAAGGAAGGGTA -3′  5′- CGTGGTGGTGAAGGAGC -3′ |
